# Chemosis as an Initial Presentation of Systemic Lupus Erythematosus

**DOI:** 10.1155/2022/4912092

**Published:** 2022-02-15

**Authors:** Carvy Floyd Luceno, Minho Yu, Daniel I. Kim, Vaneet K Sandhu

**Affiliations:** ^1^Loma Linda University Health, 11234 Anderson St, Loma Linda, CA 92354, USA; ^2^Loma Linda University, Riverside University Health Systems, 26520 Cactus Ave, Moreno Valley, CA 92555, USA; ^3^Division of Rheumatology, Loma Linda University Health, Riverside University Health Systems, 11234 Anderson St, Loma Linda, CA 92354, USA

## Abstract

Systemic lupus erythematosus (SLE) can present in a multitude of ways, which can be confounding and misleading for a clinician. Chemosis as an initial presentation is rare and has only been documented on a few case reports. However, when present, simultaneous involvement of other organs is likely. We present a previously healthy 29-year-old male who presented with severe bilateral chemosis and was subsequently diagnosed with SLE and antiphospholipid syndrome. Complications included multiple acute cerebral infarcts, lupus psychosis, lupus pleuritis, and lupus nephritis. The patient recovered well with appropriate treatment and chemosis ultimately resolved. Recognizing chemosis as an initial presentation of SLE is vital for appropriate evaluation and timely treatment to prevent disease progression.

## 1. Introduction 

SLE is a well-recognized illness with potential for devastating sequelae. Chemosis is a rare initial presenting symptom and can indicate severe underlying disease. It is important to recognize this so that workup is not delayed, and treatment is administered promptly.

## 2. Case Presentation

A previously healthy 29-year-old male presented to the hospital with vision loss. Two weeks before presentation, he experienced a subjective fever. This was followed by blurry vision with bilateral conjunctival swelling. He reported early satiety, abdominal bloating, bowel incontinence, and a weight loss of approximately 15 pounds. While in the emergency department, the patient became agitated and began banging his head against a window so that he could leave the hospital. Further history revealed that the patient had experienced a traumatic event in the past that resulted in depression, occasional paranoia, and essentially living in solitude, supported only by immediate family. Unfortunately, he never sought medical attention for his mental health and so was never diagnosed with depression or psychosis. He further denied alcohol or recreational drug use. Pertinent review of systems was negative for morning stiffness, joint pain, rash, or fatigue. He did not have any known family history of autoimmune disease.

**Figure 1:**
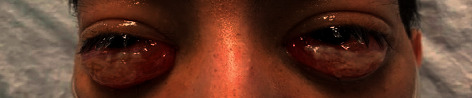
Severe inferior chemosis seen on admission.

In the emergency department, the patient was afebrile and tachycardic (88–141 beats per minute), but hemodynamically stable and on room air. He appeared thin and pale with severe chemosis of both eyes (Figure 1). Abdominal examination was significant for diffuse tenderness. Ophthalmology exam revealed chorioretinitis of both eyes and serous detachment of the left macula. The remainder of the exam was noncontributory.

**Figure 2:**
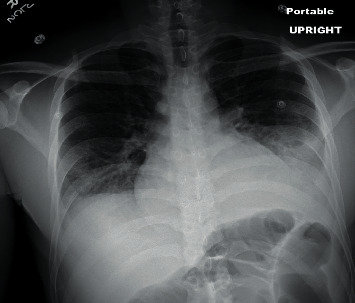
Chest X-ray revealing bilateral pleural effusions right middle lobe consolidation.

Initial labs revealed normocytic anemia (hemoglobin 11.0 g/dL), elevated creatinine (1.7 mg/dL), hypoalbuminemia (1.7 g/dL), elevated erythrocyte sedimentation rate (103 MM/hr), elevated C-reactive protein (0.8 mg/dL), elevated LDH (354 U/L), and positive antinuclear antibody (1 : 1280). Chest X-ray revealed bilateral pleural effusions (Figure 2). The patient was subsequently admitted to inpatient medicine.

Due to multisystem involvement and positive ANA on admission, a rheumatologic workup was pursued, which revealed positive double stranded DNA antibody (high titer, dsDNA 14410 IU/mL), positive anti-Sjogren syndrome-related antibody A and B (SSA, SSB), positive anticardiolipin antibody (aCL), and low complement levels.

Because of mental status changes, MRI brain with contrast was obtained and revealed multiple small acute cerebral infarcts. Due to concern for vasculitis, this was followed up by a magnetic resonance angiography of the head, which was negative. Transesophageal echocardiogram was pursued to investigate possible embolic source, but the patient was unable to tolerate the procedure due to tachycardia. A transthoracic echocardiogram showed normal left ventricular function without vegetations.

Urinalysis revealed significant protein and subsequent 24-hour urine protein was elevated (1260 mg). Renal ultrasound was negative for renal vein thrombosis. Kidney biopsy revealed class III lupus nephritis and thrombotic microangiopathy.

The patient was found to have bilateral pleural effusions and underwent thoracentesis. Pleural fluid studies were exudative (serum/pleural LDH ratio > 0.6). Due to hypoalbuminemia, the patient also developed ascites and underwent a paracentesis, which showed nonportal hypertensive ascites without evidence of inflammation. Pleural and peritoneal fluid cultures were negative for infection.

Pulse dose steroids (IV methylprednisolone 1000 mg) were initially given followed by IV methylprednisolone equivalent to 1 m/kg prednisone for concern of severe rheumatological process. Given the clinical presentation and subsequent serologic findings, the patient was diagnosed with systemic lupus erythematosus with multisystem involvement and antiphospholipid syndrome. For this, in addition to steroids, he was given cyclophosphamide 500 mg/m^2^ with mesna. He was also started on anticoagulation (warfarin with heparin bridge) due to threat of future strokes in the setting of antiphospholipid syndrome. Vision loss and chemosis were evaluated by ophthalmology, who agreed with steroids and recommended lacrilube for the chemosis and a taper of dexamethasone/neomycin eye drops. The patient was admitted for 28 days and showed marginal improvement. When he was clinically stable, he was discharged to a long-term acute care hospital for continued care.

**Figure 3:**
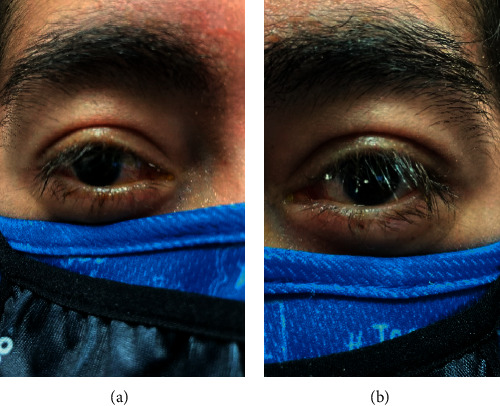
Follow up reveals resolution of severe inferior chemosis.

After one month, the patient was well enough to go home from the long-term acute care hospital. He is being followed by rheumatology and has received 4 monthly cyclophosphamide infusions. He remains on long-term steroids and was started on hydroxychloroquine, mycophenolate mofetil, and belimumab. Severe chemosis of both eyes and serous detachment of left eye have both resolved (Figure 3). At his most recent visit, he showed no signs of retinitis. He continues prednisone eye drops and lacrilube.

## 3. Discussion

Chemosis of the eye is a rare initial manifestation of lupus. In this case, chemosis was severe due to the presence of conjunctival prolapse that impaired eyelid closure [1]. One proposed mechanism is hypoalbuminemia from associated nephrotic syndrome, and another is a direct SLE-associated conjunctival inflammation. The first reported case of chemosis in SLE occurred in 1982. Zaini et al. reported a 21-year-old female in Malaysia who presented with bilateral chemosis and was subsequently diagnosed with SLE and class II glomerulonephritis [2]. In 1992, Leahey et al. reported a 36-year-old female with chemosis and anemia as the presenting signs of SLE [3]. The most recently reported case of chemosis occurred in a 35-year-old woman who presented with pressured speech and suicidal thoughts [4]. In each of the aforementioned cases, chemosis was attributed to an inflammatory process rather than to nephrotic syndrome. Other causes of chemosis include trauma, infection, allergies, postsurgery, and glaucoma [5].

The mechanism of inflammation is likely from deposition of immune complexes, like lupus nephritis. Pathology of the basement membrane of the eye epithelium in a patient with lupus and vision loss revealed immunoglobulins (IgG, IgM, IgA) and complement deposition [6] in addition to an increased number of CD4+ and CD8+ T cells, B cells, and macrophages [7, 8].

In the most recent case of reported chemosis mentioned above [4], the patient received a similar treatment course with pulse dose steroids, subsequent maintenance steroids, and monthly cyclophosphamide infusions. Like our patient, the chemosis eventually resolved. When chemosis is found, suspicion of systemic involvement should remain high as demonstrated in previous case reports. A variety of diagnostic criteria for lupus are available, with the most sensitive being the 2012 Systemic Lupus International Collaborating Clinics (SLICC) criteria [9]. When diagnosis is confirmed, treatment should be started promptly and options include hydroxychloroquine, glucocorticoids, immunomodulators, and immunosuppressants. Treatment goals are long-term patient survival, prevention of further organ damage, and quality of life optimization [10].

## Data Availability

Pertinent data used in this case report, including laboratory studies and images, are displayed and were obtained from a secure electronic medical record.
